# Altered brain glymphatic function on diffusion-tensor MRI in patients with spontaneous intracerebral hemorrhage: an exploratory study

**DOI:** 10.3389/fnagi.2024.1506980

**Published:** 2024-12-16

**Authors:** Xiaona Xia, Qingguo Ren, Juntao Zhang, Shuai Guan, Qingjun Jiang, Ying Wei, Rui Hua, Shen Zhao, Xiangjun Hu, Feng Shi, Xiangshui Meng

**Affiliations:** ^1^Department of Radiology, Qilu Hospital (Qingdao) of Shandong University, Qingdao, China; ^2^GE Healthcare PDX GMS Medical Affairs, Shanghai, China; ^3^Department of Research and Development, Shanghai United Imaging Intelligence Co., Ltd., Shanghai, China; ^4^Department of Rehabilitation Medicine, Zhongshan Hospital, Fudan University, Shanghai, China; ^5^Shanghai Baoshan District Wusong Central Hospital, Shanghai, China

**Keywords:** spontaneous intracerebral haemorrhage, glymphatic system, diffusion tensor imaging along the perivascular space, cognitive, prognosis

## Abstract

**Objectives:**

To investigate the function of the glymphatic system (GS) and its association with neuropsychological tests in spontaneous intracerebral hemorrhage (sICH) by diffusion tensor imaging analysis along the perivascular space (DTI-ALPS).

**Methods:**

This retrospective study included 58 patients with sICH and 63 age- and sex-matched healthy controls (HCs). Partial correlation analyses were performed to examine the relationships between the DTI-ALPS index and radiological as well as clinical data. Mediation analyses were performed to explore the mediating role of the grey matter proportion (GM%) in the relationship between DTI-ALPS index and Montreal Cognitive Assessment (MoCA) score.

**Results:**

Significantly lower DTI-ALPS index values were observed in sICH compared with HCs (FDR-*p* < 0.001). In the acute-subacute sICH group, the ALPS index was significantly correlated with hematoma volume (*r* = −0.572, FDR-*p* = 0.031). In the chronic sICH group, the ALPS index was significantly correlated with MoCA scores (*r* = 0.425, FDR-*p* = 0.014). In chronic sICH groups, GM% served as a significant mediator in the relationship between the DTI-ALPS index and MoCA scores (indirect effects *β* = 4.925, 95%CI: 0.028, 11.841). The ALPS index was identified as an independent prognostic indicator for unfavorable outcomes in sICH (*β* = −9.851, *p* = 0.018).

**Conclusion:**

Our study demonstrated that the DTI-ALPS index decreased in sICH patients, suggesting potential functional impairment of the lymphoid system. Additionally, the DTI-ALPS index served as an independent predictor of poor 90-day prognosis. In the acute-subacute stage of sICH, the DTI-ALPS index had negative correlation with hematoma volume. In the chronic sICH group, the GM% partially mediated the relationship between the GS and cognitive function.

## Introduction

Spontaneous intracerebral haemorrhage (sICH), which comprises about 10 to 15% of all strokes with a mortality rate of 30 to 40%, is the most serious and least treatable form of stroke (Feigin et al., 2021; Sheth, 2022). Compared with the modern systems of care for patients with acute ischaemic stroke, the lack of proven medical or surgical treatment for sICH has led to low urgency in treating these patients and a low threshold for withdrawal of proactive care, which contributes to a high rate of disability in sICH patients (Ma et al., 2023). During the 14 days following ICH, erythrolysis disrupts the blood–brain barrier, triggers inflammatory immune responses, and exacerbates hemoglobin cytotoxicity, collectively contributing to the accumulation of substantial metabolic waste, including Aβ, tau proteins, iron, and neurotoxic solutes (Ohashi et al., 2023; Peters and Lyketsos, 2023). Given the accumulation of protein aggregates is a shared feature in neurodegenerative diseases, it appears that decreased brain clearance may play a key role in neurodegeneration (Rasmussen et al., 2018). It is therefore essential for the brain to be equipped with a waste-clearing system. However, the classical waste drainage system, known as the lymphatic vessels, have long been considered absent in the central nervous system (CNS).

During the past 10 years, the “glymphatic system (GS),” has been proposed and accepted for waste clearance function in CNS (Iliff et al., 2012). The GS is a well-structured fluid delivery system, consisting of para-arterial cerebrospinal fluid (CSF) influx channels, para-venous interstitial fluid (ISF) efflux channels, and astrocyte exchange channels (aquaporin 4, AQP4) (Iliff et al., 2012). Previous studies have revealed the GS dysfunction is associated with different diseases, including demyelinating and strokes (Ji et al., 2021; Margoni et al., 2024). Early studies on glymphatic function are mostly conducted in animal models using different imaging technologies, such as two-photon imaging, fluorescence microscopy, and near-infrared imaging (Ma et al., 2019; Sweeney et al., 2019). However, none of the above technologies have been validated in human studies or have demonstrated clinical translational properties.

MRI can be applied in human research to provide dynamic, real-time images of CSF-ISF flow in the lymphoid system. Therefore, many scholars have explored the fluid dynamics and function of the lymphatic system *in vivo* in the human brain based on different models by intrathecal or administration intravenous of gadolinium (Gd)-based contrast agents (GBCAs) (Absinta et al., 2017; Zhou et al., 2020). Although GBCA-MRI is one of the most performed and validated methods for studying lymphatic system, its invasiveness and potential risk of neurotoxicity limit its clinical applicability (Naganawa et al., 2024).

Recently, a novel evaluation method known as DTI-ALPS (diffusion tensor image analysis along the perivascular space) has been proposed as a noninvasive alternative to evaluate glymphatic function (Taoka et al., 2017). Based on the fact that at the level of the lateral ventricle body, the nerve fiber bundles adjacent to the lateral ventricle are perpendicular to the medullary veins, the DTI-ALPS index provides a measure of the diffusion coefficient along the perivascular space (PVS) using a simple mathematical formula. Taoka et al., (2017) evaluated the reproducibility of the DTI-ALPS method across different scanners and found DTI-ALPS index was robust under the fixed imaging method. Although the ALPS method was theoretically deductive, a number of reports had published comparing the ALPS method with other glymphatic system evaluation methods to examine the validity of the ALPS method. Of particular importance was the study confirmed that the ALPS index was significantly related to glymphatic clearance function calculated on classically GBCA-MRI (Zhang et al., 2021). Therefore, the DTI-ALPS index may be a simple and effective image marker in assessing the clearance function of the human brain glymphoid system.

Currently, the DTI-ALPS index is proven to be strongly associated with cognitive decline in a variety of neurological disorders (Hong et al., 2024; Margoni et al., 2024). Previous animal experiments have demonstrated the alteration of GS after hemorrhagic stroke (Ding et al., 2019). To the best of our knowledge, only one article has explored altered GS function after sICH (Zhang et al., 2022). However, it only analyses the difference in ALPS scores between patients and healthy controls (HCs), as well as that between the lesion side and contralateral side with a small size of data, unexplored relationships between DTI-ALPS index with hemotoma, edema, prognosis, and cognition.

Thus, we measured the DTI-ALPS index in a cohort of sICH patients across acute to chronic phases, we hypothesized that (1) the sICH patients had impaired GS function compared to HCs; (2) the DTI-ALPS index was associated with hemotoma or edema volume, prognosis, and the cognition in sICH.

## Materials and Methods

### Participants

**Figure 1:**
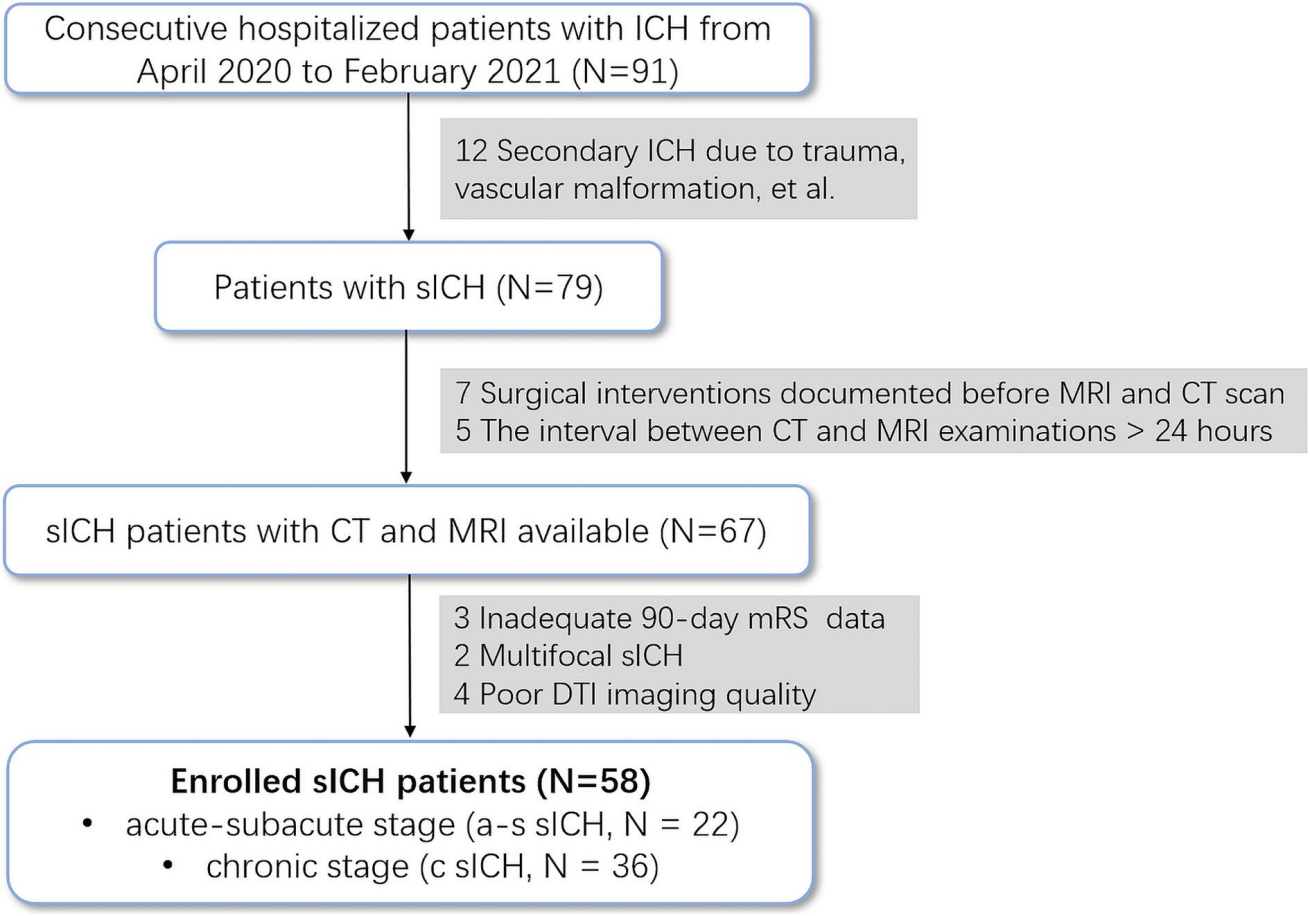
Flow diagram of the inclusion process.

We retrospectively analyzed the data on sICH patients and HCs with consecutively admitted to the acute-care stroke unit at Qilu Hospital of Shandong University (Qingdao) between April 2020 and February 2021. Considering that patients with severe intracerebral hemorrhage may have difficulty cooperating for high-quality MRI examinations, this study included only patients with mild to moderate intracerebral hemorrhage. Patients with a premorbid modified Rankin Scale (mRS) score < 2 (Xia et al., 2023), ICH volumes <60 mL (Costa et al., 2022), and Glasgow Coma Scale (GCS) scores of >6 (Costa et al., 2022) were recruited. Exclusion criteria were as follows: (I) hemorrhage secondary to factors such as trauma, vascular malformation, tumor, hemorrhagic transformation after ischemic stroke, etc.; (II) inadequate data for the 90-day modified Rankin Scale (mRS) score; (III) surgical interventions were documented before MRI and CT scan (IV)poor MRI imaging quality. To avoid human measurement errors, the automatic segmentation of intracerebral hemorrhage and edema in this study was based on CT scans. Therefore, patients with a CT and MRI examination interval greater than 24 h were excluded. Besideds, Patients with multifocal ICH were also excluded due to the complexity of analyzing hemorrhagic and edema volumes. All patients were treated according to the guideline (Hemphill et al., 2015). Demographic information, clinical data, and radiological data were documented on a standardized data collection sheet. The mRS at 12 months was assessed using a validated questionnaire by trained study staff in blinded telephone interviews. Poor functional outcome was defined as a mRS score of 3 to 6 (Hostettler et al., 2021). Figure 1 illustrates patient enrollment flow. Finally, 58 sICH patients and 63 age- and sex-matched HCs recruited from the community were included in this study. The sICH was further divided into acute-subacute stage (a-s sICH, *N* = 22) and chronic stage (c sICH, *N* = 36) with disease duration ≤3 weeks and disease duration >3 weeks, respectively.

The study has been approved by the ethics committee of Qilu Hospital. Written informed consent was waived, and the study was conducted in accordance with the Declaration of Helsinki.

### Neuropsychological assessment

All patients completed neuropsychological assessment before brain MRI examinations. Cognitive function was measured using the Montreal Cognitive Assessment (MoCA), and total MoCA scores were adjusted by years of education.

### MRI and CT acquisition and analysis

All MRI data were acquired on a 3.0 T magnetic resonance system (Philips Medical System Ingenia scanner) with a dStream head coil. The scanning sessions included: (1) three-dimensional T1-weighted imaging (3D-T1WI) for structural images of the whole brain: TR / TE = 6.7 / 3.0 ms, 170 sagittal slices, matrix = 68 × 68, 1-mm slice thickness with no gap; (2) DTI: TR / TE = 4,900 / 95 ms, matrix = 122 × 110, 70 axial slices, 2 mm slice thickness with no gap, b values = 0 and 1000s/mm^2^ with 32 gradient directions.

All CT scans were performed using Siemens Philips (Brilliance 64, Philips Medical Systems) or (SOMATOM Definition FLASH, Siemens Healthcare) scanners. The acquisition parameters were as follows: 120 kVp exposure with automatic tube current modulation technique; 1 pitch; 1 mm section thickness and matrix = 512 × 512. The reconstruction slice thickness was 5 mm.

Regions of hematoma and perihematomal edema (PHE) were detected and segmented on non-contrast CT (NCCT) using the uAI Research Portal (uRP) platform with deep convolutional neural networks (Wu et al., 2023). The grey matter (GM) and total intracranial volumes (TIV) were quantified from 3D T1 images according to the Desikan-Killiany atlas with a pre-trained cascaded VB-Nets model in the uRP tool. The grey matter proportion (%, GM%) calculated as GM/TIV was output automatically to reduce individual variances.

### DTI-ALPS processing and image analysis

After segmenting brain structures, T1 images together with brain structures were spatially realigned to DWI space via rigid registration algorithms provided by the Advanced Normalization Tools (Ants). This process facilitated the Anatomical Commissure-Posterior Commissure (AC-PC) alignment of the DTI images by using the registered brain structures in subjective DTI space. The oriented DTI images were then pre-processed with MRtrix3(Tournier et al., 2019), which included a series of steps: Marchenko-Pastur principal component analysis (MP-PCA) for denoising, correction of Gibbs-ringing artifacts, eddy current correction, and N4 bias field correction. Following these pre-processing steps, diffusivity tensors were estimated and fractional anisotropy (FA) maps were generated, and color-encoded to represent the primary diffusion directions. To calculate the DTI-ALPS index, a series of four circular regions of interest (ROIs) were positioned within the axial plane at the intersection of the corpus callosum and cerebral ventricle. Specifically, these ROIs were initially identified on a standard FA template in the Montreal Neurological Institute (MNI) space, as per the methodology established by Taoka et al. (2022). Subsequently, the FA template was registered to the DTI space to ascertain the initial coordinates for the ROIs. An in-house algorithm was then employed to automatically adjust the positioning of the projection and association peaks within the color-encoded FA maps, localized to both sides of the mid-posterior corpus callosum. Finally, ROIs with a 3 mm radius were delineated, centered on these peaks. The diffusivity within these ROIs was quantified along the x-axis ($D_{x}$), y-axis ($D_{y}$), and z-axis ($D_{z}$) for both projection fibers ($D_{\mathrm{xproj}}$, $D_{\mathrm{yproj}}$,$D_{\mathrm{zproj}}$) and association fibers ($D_{\mathrm{xassoc}}$, $D_{\mathrm{yassoc}}$, $D_{\mathrm{zassoc}}$), respectively. Then the ALPS index was calculated as follows (Taoka et al., 2017):

$$\mathrm{ALPS index}=\frac{\mathrm{mean}\left(D_{\mathrm{xassoc}},D_{\mathrm{xproj}}\right)}{\mathrm{mean}\left(D_{\mathrm{zassoc}},\;D_{\mathrm{yproj}}\right)}$$

**Figure 2:**
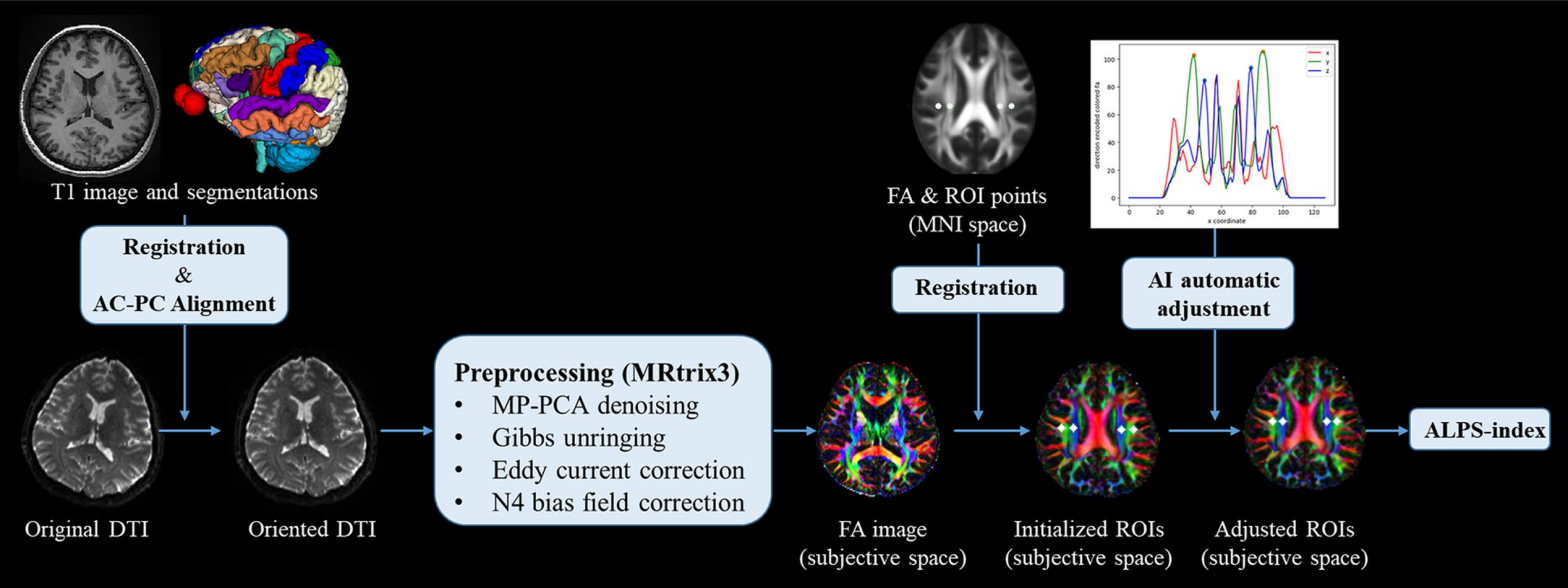
Workflow of DTI processing and ALPS index calculation.

The workflow of DTI data processing and ALPS index calculation is summarized in Figure 2. The mean value of left and right ALPS index was used in the HCs group, and the ALPS index of the lesion side was used in the sICH group.

### Statistical analysis

Statistical analyses were performed on SPSS software version 22 (IBM Corporation) and in R (version 4.3.3). The normal distribution of continuous variables was detected by the Shapiro–Wilk test. Continuous variables were expressed as mean ± standard deviation or median [interquartile range, IQR], and categories as number [percentage (%)]. Independent samples t-test and Wilcoxon rank sum test were performed for continuous variables for between-group comparisons, while Chi-squared analysis for categorical variables. To analyze DTI-ALPS index differences between sICH patients and HCs, a general linear model was constructed to correct for possible confounders (including age, sex, hypertension, diabetes, and TIV) by propensity score matching.

Partial correlation analyses were performed between the DTI-ALPS index with radiological and clinical data in ICH patients. We employed the Spearman method for the relationship between MoCA scores with ALPS indices. In contrast, we utilized the Pearson method for the relationship between hematoma and PHE volumes with ALPS indices. Age, sex, and TIV were set as covariates. The false discovery rate (FDR) correction method was applied to correct for multiple comparisons. A bilateral *P* < 0.05 was deemed statistically significant. Multivariable logistic model with the backwards elimination procedure was constructed using mRS as the dependent variable to explore whether ALPS index was an independent predictive factor with adjustment for relevant confounding variables.

Mediation analyses were carried out to explore the mediating role of GM% on the relationship between DTI-ALPS and MoCA score. Detailly, the mediation analysis was performed by PROCESS for SPSS. A 95% bootstrap confidence interval based on 5,000 bootstrap replicates was used to estimate significance.

## Results

### Demographic, clinical and conventional MRI findings

Table 1 summarizes the main demographic and clinical data in sICH patients and HCs. Finally, 58 sICH patients (35 males and 23 females, 53.8 ± 11.7 years old) and 63 HCs (35 males and 28 females, 54.6 ± 8.3 years old) were included in the present study. Only hypertension showed significant difference between sICH and HC groups (*p* < 0.001), while no significant differences were found in age, sex and diabetes (all *p* > 0.05). Apart from disease duration, no significant differences were observed in any of the other variables between a-s sICH and c sICH groups (all *p* > 0.05).

**Table 1:** Demographic and clinical characteristics of HCs and sICH patients.

Variable	HCs (*n* = 63)	sICH (*n* = 58)	*p* value	a-s sICH (*n* = 22)	c sICH (*n* = 36)	*P* value
Male, *n* (%)	35(55.6)	35(60.3)	0.594^a^	12 (54.5)	23 (63.9)	0.480^a^
Age (years)	54.6 ± 8.3	53.8 ± 11.7	0.699^b^	55.1 ± 8.5	53.1 ± 13.3	0.488^b^
GCS	-	12.0 [10.0;14.0]	-	12.0 [10.0;14.0]	-	-
Diabetes, *n* (%)	5 (7.9)	10 (17.2)	0.121^a^	4 (18.2)	6 (16.7)	1.000^a^
Hypertension, *n* (%)	17 (27.0)	50 (86.2)	<0.001^a^	19 (86.4)	31 (86.1)	1.000^a^
Hematoma volume (ml)	-	-	-	13.0 [6.6;18.0]	-	-
PHE volume (ml)	-	-	-	20.1 [9.9;30.0]	-	-
Disease duration (days)				5.0 [3.0;9.0]	210.0 [97.5;729.0]	<0.001^c^
mRS > 2, *n* (%)	-	14 (24.1)	-	6 (27.3)	8 (22.2)	0.663^a^
MoCA	-	27.0 [21.0;29.0]	-	25.5 [21.0;29.0]	28.0 [20.5;29.0]	0.407^c^

^aChi-square test; btwo sample t-test; cWilcoxon test.^

### DTI-ALPS index

**Figure 3:**
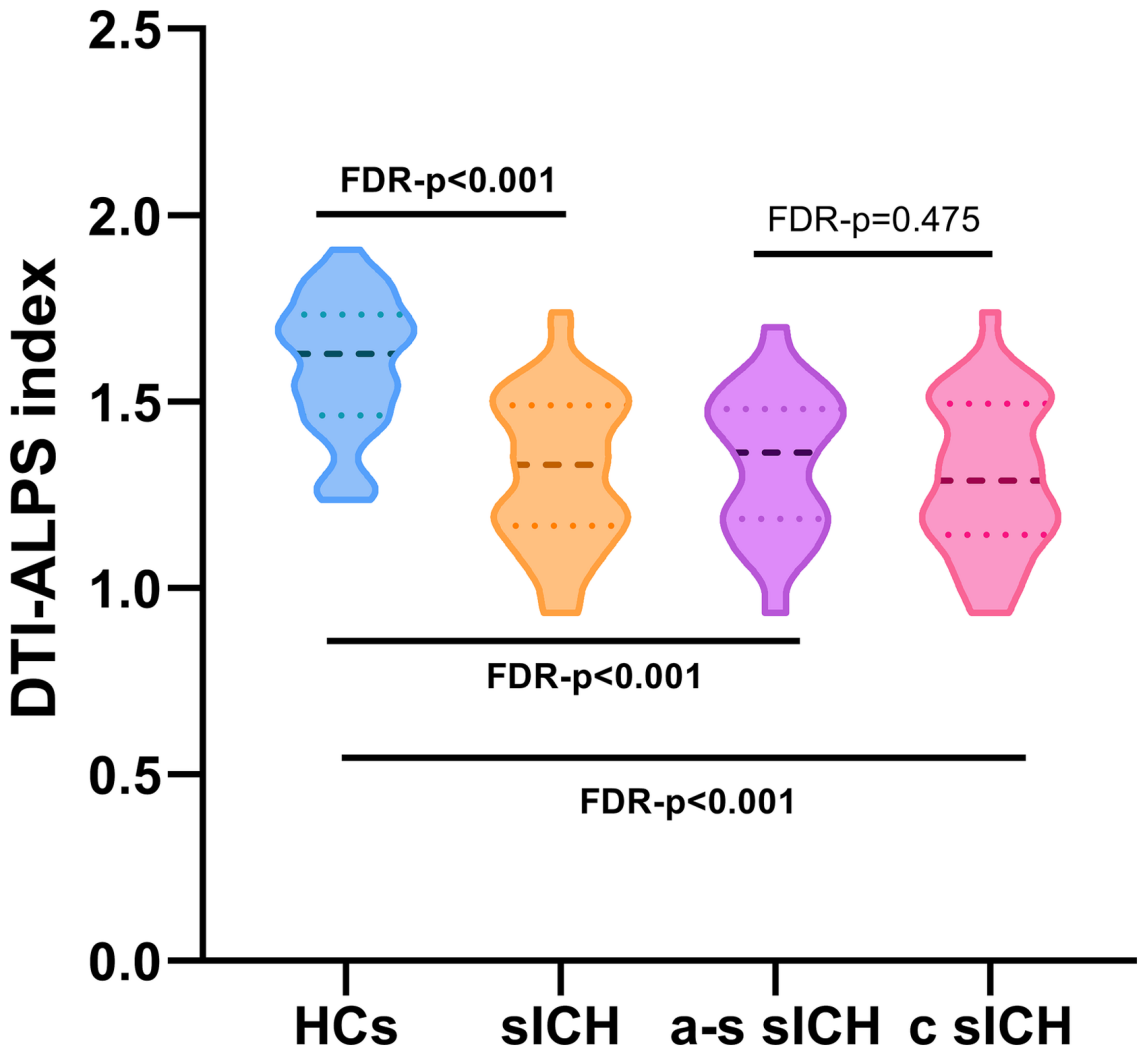
The DTI-ALPS index of sICH patients and HCs.

In this study, compared with HCs, sICH patients had a significantly lower ALPS index after correcting for possible confounders (including age, sex, hypertension, diabetes and TIV) (FDR -*p* < 0.001). No significant difference was found in ALPS index between a-s sICH and c sICH groups (FDR-*p* = 0.475) (Figure 3).

### Partial correlation analyses

**Figure 4:**
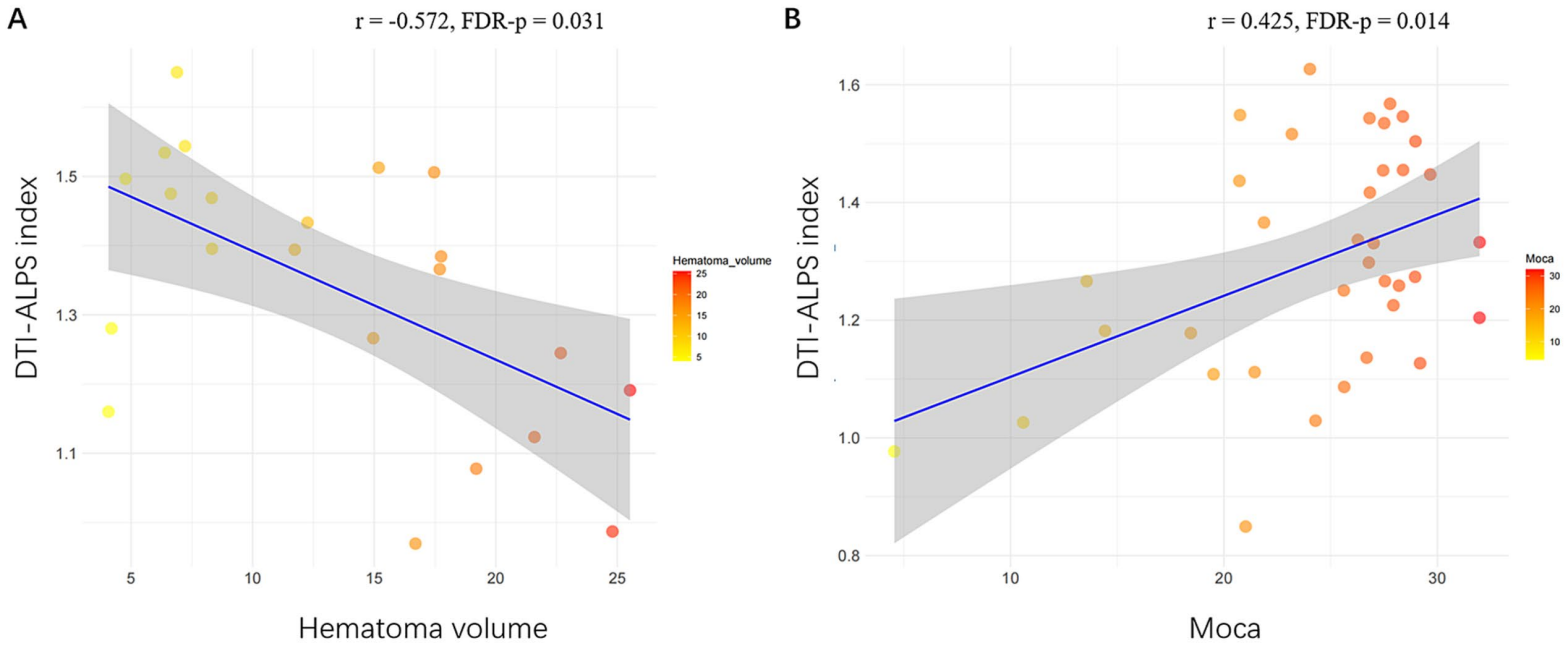
Significant associations of DTI-ALPS index with radiological and clinical data in sICH patients. **(A)** Scatterplots show significant association of larger ALPS index with smaller hematoma volume volume. **(B)** Scatterplots show significant association of larger ALPS index with higher MoCA score in c sICH group. sICH, spontaneous intracerebral haemorrhage; MoCA, Montreal Cognitive Assessment; c sICH, chronic stage sICH.

In sICH patients, the ALPS index was significantly positively correlated with the MoCA scores (*r* = 0.393, FDR-*p* = 0.009). In a-s sICH patients group, no significant association was observed between DTI-ALPS index and MoCA scores (FDR-*p* = 0.365). Besides, DTI-ALPS index showed significant association with hematoma volume (*r* = −0.572, FDR-*p* = 0.031) while no significant association with PHE volume after FDR correction (*r* = −0.461, FDR-*p* = 0.071). In c sICH patients group, the ALPS index was significantly correlated with the MoCA scores (*r* = 0.425, FDR-*p* = 0.014) (Table 2 and Figure 4).

**Table 2:** The association between DTI-ALPS and clinical data in different groups.

Variable	DTI-ALPS					
	sICH group		a-s sICH group		c sICH group	
	*r*	FDR-p	*r*	FDR-p	*r*	FDR-p
Hematoma volume	-	-	−0.572	0.031	-	-
PHE volume	-	-	−0.461	0.071	-	-
MoCA score	0.393	0.009	0.365	0.220	0.425	0.014

### Mediation analyses

**Figure 5:**
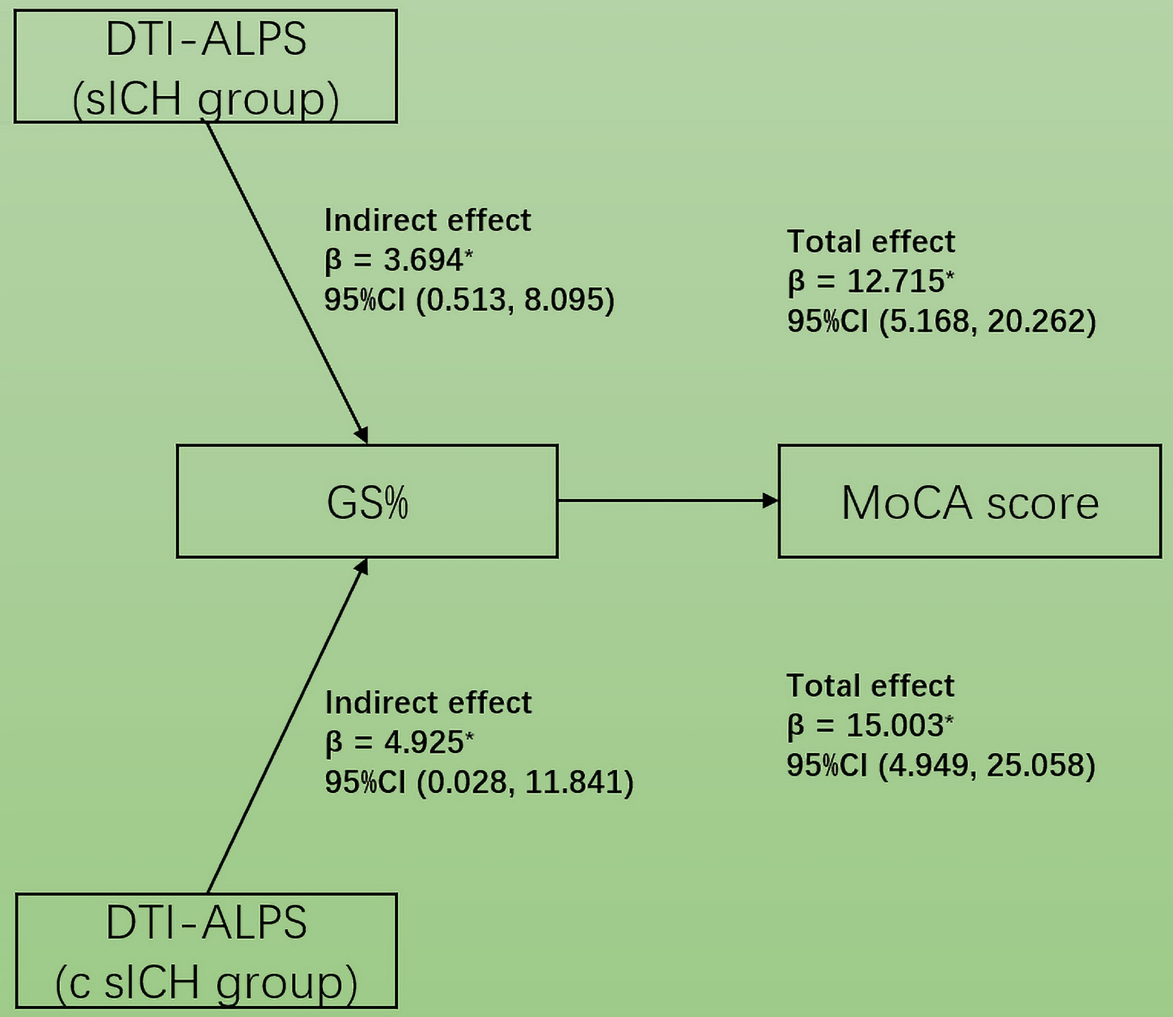
Simple mediation diagram in sICH patients. The GM propotion mediated the association between DTI-ALPS index and MoCA score in both sICH and c sICH groups.

In the mediation analysis, the MoCA score was affected by the DTI-ALPS index via GM% both in sICH and c sICH groups (indirect effects *β* = 3.694, 95%CI: 0.513, 8.095 and *β* = 4.925, 95%CI: 0.028, 11.841) (Figure 5). The changes in GM% aligned with those in both the ALPS index and MoCA scores.

### DTI-ALPS index and its diagnostic value in prognosis for sICH

**Figure 6:**
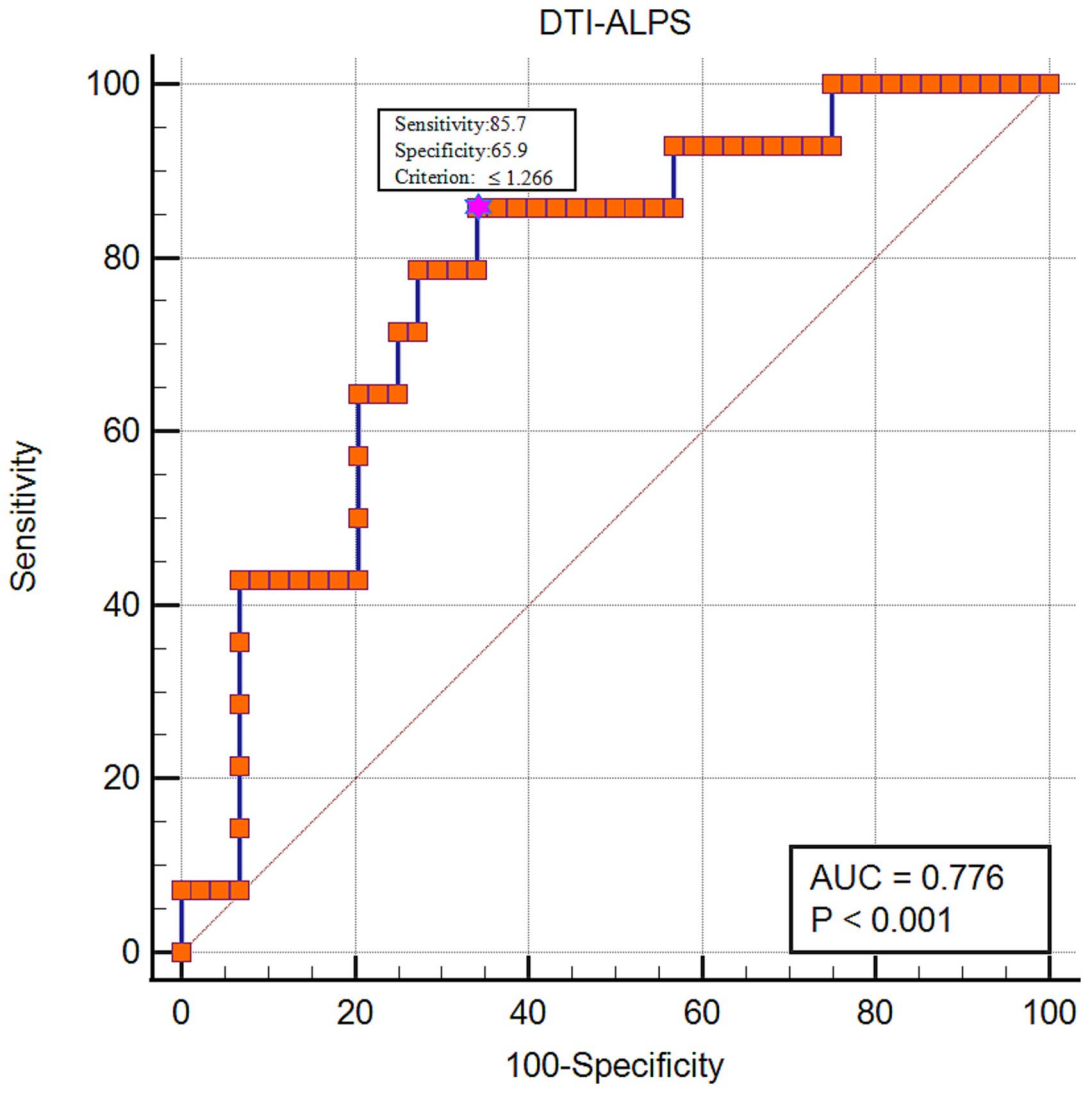
The diagnostic performance of the DTI-ALPS index in predicting 90-day poor functional outcome for sICH patients.

The multivariable logistic model established with including age, sex, GCS, hypertension, diabetes, Moca score, hematoma volume, PHE volume and ALPS index as predictive factors showed that ALPS index was an independent predictor of 90-day poor outcome for sICH (*β* = −9.851, *p* = 0.018). ROC curve analysis was further performed to assess the performance of the ALPS index for predicting sICH poor outcome, and the area under the curve (AUC) for the ALPS index was 0.776 (95% CI: 0.647–0.875, *p* < 0.001). The cut-off value was 1.266, the sensitivity was 0.857, and the specificity was 0.659 (Figure 6).

## Discussion

To our best knowledge, our study is the first attempt to explore glymphatic system function in sICH stratified according to disease duration and its correlation with clinical prognosis and cognition impairment *in vivo* by the DTI-ALPS method. Our study demonstrated a decrease in DTI-ALPS index, and changes in this measure were seen under conditions where GS function was impaired (Taoka et al., 2024). This result suggested that patients with sICH may have impaired GS function.The DTI-ALPS index was negatively correlated to hematoma volume. In addition, the DTI-ALPS index was significantly associated with cognitive performance (measured by MoCA score) in c sICH group and their relationship was mediated by GM proportion.

Consistent with our hypothesis, sICH patients and its subgrous all exhibited decreased DTI-ALPS indexes compared with HCs, indicating an impaired brain GS function, which was in accordance with previous research finding (Zhang et al., 2022). Liu et al. (2023) observed the ICH group had an impaired glymphatic system, which provided additional animal experimental support to our research. Previous studies had confirmed that a serious pathophysiological changes occurred after cerebral hemorrhage including blood–brain barrier dysfunction, inflammatory immune response, and red blood cell lysis (Wan et al., 2023), leading to immune cell dysfunction, accumulation of metabolic wastes, and dislocalization of AQP4 (Ding et al., 2019; Lan et al., 2019), which further resulted in the dysfunction of GS and hydrocephalus (Chen et al., 2021). Following ICH, the loss of aquaporin-4 polarity was coupled with the loss of *β*-dystroglycan in the perihematomal area(Qiu et al., 2015). In an ICH model of rats, GS dysfunction exacerbated cerebral edema, neuroinflammation, neuronal apoptosis and caused neurological deficits via down-regulating AQP4, upregulating inflammatory TNF-*α* and inhibiting IL-10 expression(Liu et al., 2021). However, the role of the GS system in intraparenchymal hematoma/erythrocyte clearance and ICH pathology remains incompletely understood and warrants further basic research. In contrast to the previously reported positive feedback mechanism between GS function and disease progression (Rasmussen et al., 2018), our research revealed that GS did not exhibit a consistent decline throughout the course of sICH since no significant difference was found in ALPS index between a-s sICH and c sICH groups. It was speculated that this finding may be related to factors such as the relatively small hemorrhage volume and clinical interventions in our study. Future studies with larger sample sizes are needed to further investigate the changes in GS characteristics at different stages of sICH with varying hemorrhage volumes.

In this study, the DTI-ALPS index was significantly associated with hematoma volume in a-s sICH group, which was an interesting finding and had not been reported in other literature as far as we investigated. Our finding may be partly supported by the previous result that enlarged PVSs both in basal ganglia and centrum semiovale were seen in ICH patients (Wang et al., 2019). This phenomenon may be explained by the fact that larger hematoma may trigger more severe immune-inflammatory response, causing more neuronal apoptosis and hemoglobin degradation, producing more metabolic waste products and thus causing more severe GS dysfunction (Chen et al., 2020), which should be further validated in basic experiments.

ICH survivors were at high risk of cognitive decline following stroke onset with cognitive impairment incidence ranging from 14.2–77% due to population diversity, diagnostic criteria, stage of ICH, regions, and other factors (Garcia et al., 2013; Moulin et al., 2016). This study demonstrated a lower MoCA score in a-s sICH group compared to the c sICH group, in accordance with the previous findings that cognitive impairment rate was relatively high during the acute phase and dropped off over the first 2 years post-ICH (Potter et al., 2021). The underlying mechanisms of post-ICH cognitive impairment are not known in detail. Cognitive impairment in the acute post-ICH phase was likely to be related to multiple factors such as acute neurological injury, condition at admission, ICH location, etal, (Potter et al., 2021), which may be the reason why GS was not significantly correlated with MoCA score in the a-s sICH group in our study. In c sICH patients group, the ALPS index was significantly correlated with the MoCA score, indicating the metabolic waste accumulation due to GS dysfunction played a role in the development of cognitive decline in chronic post-ICH phase, similar to other neurodegenerative diseases (Burfeind et al., 2017). The relationship between GM reductions and cognition impairment was proven in a variety of neurological disorders including hemorrhagic stroke (Krajcovicova et al., 2019; Wang et al., 2023). Our mediation analysis suggested that GS dysfunction may lead to cognitive impairment, partially mediated by global GM damage after sICH. In this regard, DTI-ALPS may represent a novel neuroimaging biomarker for cognitive impairment following sICH.

Furtherly, our findings suggested that DTI-ALPS index showed good performance in predicting 90-day poor prognosis for sICH. Studies about the relation between ICH outcome and GS function were limited and mostly focused on the role of dilated PVS in ICH. Previous research demonstrated that dilated PVS was associated with stroke recurrence or amyloid deposition (Tsai et al., 2021, 2022), both of which were related to the poor prognosis in ICH. Importantly, given the complex impacts of GS on multiple aspects of ICH development, we suggest that early improvement of GS function may be a potential strategy for better functional outcome in sICH patients. Thus, our findings may have an important role in exploring novel therapeutic directions for ICH.

Inevitably, our study has several limitations. First, this was a single-center study with a relatively small sample size, necessitating an expanded sample size for a longitudinal study design to explore the role of GS in the development of ICH. Second, due to the difficulty in the enrolment of severe ICH subjects in MRI studies, only mild to moderate ICH patientis were recruited, potentially limiting the extent of variability of DTI-ALPS index in ICH. Third, the neuropsychological assessments for HCs were absent, although partial correlation analyses were performed with age, sex, and TIV as covariates to explore the relationship between DTI-ALPS index with MoCA score. Since pre-existing neurocognitive disorders prior to ICH are associated with an increased risk of poor outcomes (Zuo et al., 2024), baseline MoCA scores may be an important confounding factor in the analysis of intracerebral hemorrhage prognosis. Therefore, the relationship between the DTI-ALPS index and the prognosis of sICH needs to be further validated through more rigorous experiments that include baseline cognitive function. Forth, we did not exclude potential impact of confounders such as cerebral small vessel diseases, subarachnoid hemorrhage and ICH location, which might have some interactions with GS activity and neuropsychological assessments (Li et al., 2024). Fourth, the DTI- ALPS index could be measured only in the axial plane of the lateral ventricle body, where often be involved by hematoma, which may affect the accuracy of DTI-ALPS index. Besides, the DTI-ALPS index method is a direction-dependent analysis with mixing of different information including the surrounding white matter (Taoka et al., 2024), although study had confirmed that the ALPS index was significantly related to GS function calculated on gold standard - GBCA MRI (Zhang et al., 2021). Therefore, innovate methods should be investigated to measure glymphatic activity in the other region of brain to explore regional glymphatic function in the future. In mild to moderate sICH, process of care related factors have strong impact on mortality (Fernandes et al., 2022). Although all patients in this study were admitted to acute-care stroke units and managed according to clinical guidelines, future research on ICH prognosis should explore the impact of care-related factors.

## Conclusion

The DTI-ALPS index was decreased in sICH, suggesting possible impaired function of the GS system. The DTI-ALPS index served as an independent predictor of poor prognosis for sICH in this study, indicating impaired GS function may be associated with the poor prognosis of patients with sICH. Additionally, the DTI-ALPS index was negatively correlated with hematoma volume in the acute sICH patient group. Furthermore, a decrease in the DTI-ALPS index may lead to cognitive impairment, partially mediated by GM proportion in the chronic stage of sICH. Our study provides ancillary support for the important role of GS dysfunction in sICH pathology and suggests that early enhancement of glymphatic function may represent a promising therapeutic approach to optimize prognosis in sICH.

## Data Availability

The raw data supporting the conclusions of this article will be made available by the authors, without undue reservation.
